# Ultrasound‐modified whey protein‐enriched instant noodles: Enhancement in functional, rheological, cooking, and structural attributes

**DOI:** 10.1002/fsn3.3797

**Published:** 2023-11-06

**Authors:** Anju Boora Khatkar, Amarjeet Kaur, Sanju Bala Dhull, Sunil Kumar Khatkar, Nitin Mehta, Jaspreet Kaur, Gulden Goksen

**Affiliations:** ^1^ Department of Food Science & Technology Punjab Agricultural University Ludhiana Punjab India; ^2^ Department of Dairy Chemistry, College of Dairy Science and Technology Guru Angad Dev Veterinary and Animal Sciences University Ludhiana Punjab India; ^3^ Department of Food Science and Technology Chaudhary Devi Lal University Sirsa Haryana India; ^4^ By‐products Utilization Lab, Department of Dairy Technology, College of Dairy Science and Technology Guru Angad Dev Veterinary and Animal Sciences University Ludhiana Punjab India; ^5^ Department of Livestock Products Technology, College of Veterinary Science Guru Angad Dev Veterinary and Animal Sciences University Ludhiana Punjab India; ^6^ Department of Food Technology, Vocational School of Technical Sciences at Mersin Tarsus Organized Industrial Zone Tarsus University Mersin Turkey

**Keywords:** cooking quality, instant noodles, ultrasound, whey protein

## Abstract

Instant noodles enriched with ultrasound‐modified whey protein (WP) were characterized for physical, technological, rheological, cooking, thermal, in vitro protein digestibility (*IVPD*), morphological, and sensory attributes to access the compatibility of ultrasound for actual food matrix. Semolina with sonicated WP (SWP) showed significantly (*p* < .05) higher water absorption capacity (1.586 g/g) than semolina with raw WP (1.512 g/g). Semolina with SWP also showed a significantly higher water solubility index, oil binding, and firmer gel, even at 5% concentration. The addition of SWP positively impacted pasting properties and improved dough handling, as also supported by the constantly increasing storage (*G*′) and loss (*G*″) modulus. SWP significantly decreased the cooking loss (7.48%) and considerably increased cooking weight (13.80%), water uptake ratio (14.35%), noodle diameter, expansion (4.47%), hardness, springiness, gumminess, and chewiness of instant noodles. Thus, the instant noodles containing SWP imparted high resistance to tear and wear to noodle strands. The improved thermal, *IVPD* (90.46%), and acceptability with excellent structural (morphology) integrity authenticate SWP as a quality protein source for enrichment.

## Graphical abstract

SWP‐enriched instant noodles exhibited better cooking, textural, in vitro *digestibility* and overall acceptability. Morphology also validated the structural strength and integrity of instant noodles. Study offers a scientific solution for complication linked to protein enrichment

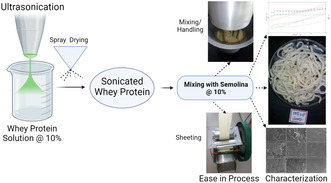

## 1 INTRODUCTION

Instant noodles, one of the main wheat‐based products, are among the most popular dietary items. At the global level (WINA, 2020), around 106.4 billion instant noodles were consumed in 2019; of these, more than 80% were by Asians. The consumers' interest in affordability, convenience, appearance, taste, and satisfying texture are the driving factors. However, the noodle market is dominated by wheat‐based fried noodles, which are primarily starchy and lower in quality protein. They are deficient in lysine and threonine, and as a result, they cannot meet a person's daily requirement for quality protein. In developing countries, the prevalence of perceived protein energy malnutrition (PEM) is crucial as it leads to deteriorating health and functioning, increasing susceptibility to infection, depression, and even silent death. Unfortunately, doctors rarely diagnose it, and even when they do, they hardly ever recommend or provide treatment. Simultaneously, the demand for health and quality nutrition is soaring due to modernization and the current pandemic. As a result, it is prioritized as one of the most challenging fields. Therefore, it is vital to provide technological solutions to enrich quality protein in convenient foods, such as instant noodles, to satisfy the need for protein and energy. Several researchers have targeted instant noodles to improve the protein profile like yellow‐peas, chickpeas, and lentils flour (Bouasla et al., 2017); faba flour (Laleg et al., 2017); lupin flour (Mahmoud et al., 2012); fish powder (Desai et al., 2018); soy protein (Khatkar et al., 2021; Khatkar & Kaur, 2018) and egg albumin, whey protein, and rice bran protein (Phongthai et al., 2017). But a sustainable and viable concept for the food industry is adding quality protein, such as whey protein, into food systems without compromising the quality and functionality. Whey protein (WP) has been endorsed as a food ingredient with many health advantages for its immune‐boosting properties and biological components for anti‐inflammatory, anti‐tumor, anti‐hypertensive, hypolipidemic, antiviral, antibacterial, and chelation (Khatkar et al., 2018b). However, the food industry is also experiencing technical difficulties in handling WP and other proteins, particularly regarding the stability, sensory, and textural qualities.

Furthermore, adding protein to flour makes the dough more robust and challenging to handle and machinate (Khatkar et al., 2021; Khatkar & Kaur, 2018). As a result, some modification strategies are needed to tailor the proteins for better suitability and functionality. Ultrasound has excellent potential for modifying the functionality of many proteins, including WP, without any negative impact (Khatkar et al., 2018a, 2018b, 2020, 2021; Siewe et al., 2021). The technique is considered non‐toxic, safe, and environmentally friendly; and has already caught the industry and academia's attention. However, its products are not explored much in the actual food matrix as the industry is still looking for more supportive data for its adoption. Therefore, the present study was envisioned to evaluate the impact of the addition of ultrasound‐modified WP on various quality attributes of instant noodles. The findings will have wider applications in the food and dairy industry wherein protein fortification strategies can be applied effectively for the development of protein fortified foods. Additionally, high‐protein foods will be better maintained at higher processing temperatures with desired overall quality attributes.

## 2 MATERIALS AND METHODS

### 2.1 Raw materials

Medium‐coarse size (350–375 μm) semolina (Kumar Food Industries Limited – Shakti Bhog, Siraspur, Delhi) having 14.50 ± 0.07% moisture and 9.98 ± 0.08% protein, and salt (R.K. Buffer, Alamgir, Ludhiana) of Tata brand were procured from the local market. The WP concentrate (WPC‐80) Fonterra (New Zealand) was provided by Sindhwani Traders India, New Delhi. The sample preparation and subsequent analysis were performed with Milli‐Q water.

### 2.2 Noodles preparation

For protein fortification, WP was modified as described in our previous work (Khatkar et al., 2018a) with Sonics‐VCX 750 (Vibra Cell Sonics; Sonics & Materials Inc). The study involved subjecting WP solution (12.75% concentration) to ultrasound at a frequency and amplitude of 20 kHz and 20%, respectively, for 19.75 min. Subsequently, the resulting sonicated solution was subjected to spray drying using a Mini Spray Dryer (B‐290; BUCHI India Pvt. Ltd) while maintaining inlet and outlet air temperatures at 185 ± 2°C and 85 ± 2°C, respectively, to obtain SWP. After that, the control and protein‐enriched (raw WP and SWP added 10%) instant noodles were prepared following the sheeting method as described by Khatkar and Kaur (2018) with slight modifications. The semolina (as control), semolina with raw WP (RWP), and semolina with SWP, were hydrated with 33%, 28%, and 31% water, respectively, based on their different water hydration capacities. The formulations were adequately hydrated and homogeneously blended (17 min) using a dough mixer. The resulting crumbled dough was shaped into a spherical mass and subsequently enclosed within plastic pouches for 30 min at room temperature. Subsequently, the dough mass underwent manual kneading for 1 min, and then it was rolled into sheets using a dough sheeter (SH‐E‐L Pat Pend National Manufacturing Company, Lincoln, NEBR.‐68508). The process of dough sheet formation involved repetitive (7–8 times) folding and sheeting, while concurrently adjusting the roller gap to gradually attain the desired thickness of around 1 mm. The dough sheets were slit by passage through a noodle machine (KSC), followed by a steaming procedure lasting 10 min. The resulting steamed noodles were subsequently subject to drying within a tray dryer (set at 50°C) until reaching the intended moisture content of 5%–7%. After cooling to room temperature, the dehydrated instant noodles were hermetically enclosed in polyethylene bags and stored within a controlled environment of 12–14°C until subjected to analysis.

### 2.3 Evaluation and characterization

The raw semolina and protein‐enriched formulations for instant noodles were assessed for physical (density and dispersibility), technological properties such as water absorption capacity (WAC, g/g), water solubility index (WSI, %), oil absorption capacity (OAC, %) and least gelation capacity (LGC, %), and pasting features. The instant noodles were assessed for dimensional properties (noodles diameter, mm and expansion, %); cooking (time, min; loss, %; weigh, %; water uptake ratio; and swelling index, %) qualities; texture; instrumental color; thermal (*T*_o_, onset; *T*_p_, peak; *T*_e_, end set temperatures; and enthalpy, Δ*H*); invitro protein digestibility (*IVPD*, %) as per the methods described by Khatkar and Kaur (2018).

### 2.4 Dough rheology

The dough sheet rheology was assessed using the MCR 301 rheometer (Anton Paar, GmbH) equipped with Rheoplus 32 v2.81 software. The rheological measurements were conducted with a 2‐mm gap maintained between parallel plate geometry (PP25 – SN24208). Before the examination, the raw material(s) were hydrated with a calculated amount of water for 15 min, and then sheets (9‐fold sheeting) were prepared. The extra outer exposed portion of the dough sheet between the plates was finely cut and covered with oil (paraffin) to prevent dehydration. The dough was allowed to rest for 5 min at 25°C before the examination. The linear viscoelastic (LV) limits were determined initially using a strain sweep test, with the strain set at 0.01 within the LV range. Subsequently, a frequency sweep was conducted over the 0.1–10 Hz range to determine the dough's storage modulus (*G*′) and loss modulus (*G*″).

### 2.5 The surface morphology of instant noodles

The uncooked instant noodles samples were placed on a black plate and coated with gold using a vacuum evaporator. Subsequently, the black plate with the samples was secured in a Hitachi S–3400 N Scanning Electron Microscope. Images were captured at magnifications of 500× and 100× to assess the samples' structural morphology and integrity (Khatkar & Kaur, 2018).

### 2.6 Sensory evaluation

Throughout the study, a semi‐trained panel of judges consisting of seven faculties and scholars from the Department evaluated the sensory parameters, that is, surface appeal, hardness, stickiness, biting behavior, mouthfeel, and overall acceptability of cooked instant noodles. The assessment was conducted using a 9‐point hedonic scale, ranging from 1 (disliked extremely) to 9 (liked extremely). The noodles were cooked in boiling water (1 L) for their recommended cooking time, and the cooked samples (100 g) were then analyzed for various sensory attributes.

### 2.7 Statistical analysis

The descriptive statistics and the general linear model's full factorial model of multivariant components employing IBM SPSS Statistics (22.0 version) were used for data analysis. The post hoc multiple comparisons and Duncan's multiplication test (*p* < .05) were used for observed means and equal variance.

## 3 RESULTS AND DISCUSSION

### 3.1 Physical and technological properties

The addition of SWP (sonicated whey protein) significantly (*p* < .05) improved the WAC of semolina compared to the semolina containing raw WP (RWP) (Table 1). However, both the RWP‐ (1.512 g/g) and SWP (1.586 g/g)‐fortified semolina samples exhibited lesser WAC compared to the earlier reported values (Khatkar et al., 2018a, 2018b) for raw semolina. Sudha et al. (2011) and Porwal et al. (2014) observed similar behavior for WAC for WP in wheat flour and chickpea flour, respectively. The increase in blend WAC by SWP compared to the RWP indicates that sonication increases the ability of WP to interact with water‐exposing functional buried groups. The WSI of RWP and SWP formulations had no significant (*p* > .05) difference but SWP incorporation showed slightly higher WSI (11.83%) than RWP (11.60%) containing semolina. The formulations containing raw and sonicated WP showed >3× higher WSI compared to earlier reported values of raw semolina which might be due to the replacement of semolina proportion with highly soluble WP in addition to the effect of sonication that increases the WP's solubility (Khatkar et al., 2018a); thus, also enhances the formulation's WSI. Bouasla et al. (2017) also reported increased WSI by incorporating lentil flour, yellow peas, and chickpeas in gluten‐free pasta. The SWP containing semolina had significantly (*p* < .05) higher OAC. Proteins are amphiphilic and contain both hydrophilic and hydrophobic groups. These groups, particularly hydrophobic ones, make it easier for proteins to interact with oil and increase the flour's ability to bind oil. Due to conformational changes by ultrasonic treatment, suppressed hydrophobic groups of protein become accessible, which increases the SWP formulation’ OAC (2.28%). The results for OAC are consistent with what Khatkar et al. (2021) reported for restructured soy protein in instant noodles. SWP blend exhibited significantly (*p* < .05) lower bulk density (0.72 g/mL) than the RWP (0.84 g/mL) blend. The SWP blend's lower bulk density was due to its reduced particle size. There were no significant (*p* > .05) differences in the dispersibility of semolina containing RWP and SWP. The lower LGC values represented better gelation capacity, and the formulation with SWP formed a firmer gel, even at 5% (Table 1), than RWP (6%) containing semolina gel. The gelation results were better than the restructured soy protein‐enriched semolina, as Khatkar et al. (2021) reported. Protein gelation was significantly affected by the exposed hydrophobic and sulfhydryl groups of proteins. Ultrasonic cavitation induces the unfolding of WP's helical structure and promotes intermolecular hydrophobic interaction and conformational variations (Zisu et al., 2011).

**TABLE 1:** Physical, technological, and pasting properties of SWP‐enriched semolina vis‐à‐vis RWP‐enriched semolina.

	Physical and technological properties					Pasting properties					LGC (%)				
	WAI (g/g)	WSI (%)	OAC (%)	BD (g/mL)	Dis. (%)	Peak viscosity	Hold viscosity	Final viscosity	Break‐down viscosity	Setback viscosity	5	6	7	8	9
RWP + Semolina	1.512 ± 0.005^a^	11.60 ± 0.3^a^	2.00 ± 0.78^a^	0.84 ± 0.01^b^	82.08 ± 0.14^a^	1211.67 ± 20.82^b^	1178.67 ± 18.58^b^	3289.33 ± 27.59^b^	29.67 ± 1.15^b^	2117.33 ± 29.69^b^	−	+	+	++	++
SWP + Semolina	1.586 ± 0.017^b^	11.83 ± 0.06^a^	2.28 ± 0.01^b^	0.72 ± 0.01^a^	82.00 ± 0.87^a^	1165.33 ± 11.37^a^	1136 ± 18.33^a^	3175.67 ± 78.01^a^	23.67 ± 0.58^a^	2020.33 ± 28.18^a^	+	++	++	++	++

*Note*: Means (*n* = 3) with the same superscript in a column are not significantly different *p* < .05 from each other. RWP, SWP, WAI, WSI, OAC, BD, Dis, and LGC are the raw whey protein, sonicated whey protein, water absorption index, water solubility index, oil absorption capacity, bulk density, dispersibility, and least gel concentration (− not gelled, ± slightly set, + set, ++ fully set), respectively.

### 3.2 Pasting properties

SWP significantly (*p* < .05) reduced the pasting characteristics (Table 1) of semolina than the RWP, which might be attributed to the thinning and particle size reduction effect of ultrasound. However, the semolina containing RWP and SWP exhibited lesser pasting properties than the reported values for semolina alone (Khatkar et al., 2018a, 2018b) due to the proportionate dilution and substitution of starch concentration with protein(s). Starch typically forms a more viscous gel when the concentration is higher than that of the protein. The peak, hold, final, breakdown, and setback viscosities were higher for RWP blends than the SWP blends. Peak viscosity, also linked to WAC and the finished product quality, demonstrates the starch's capacity to swell freely before starch's physical and structural breakdown (Adebowale et al., 2005). SWP's higher solubility and smaller particle size also contributed to a significant (*p* < .05) decrease in peak viscosity. Khatkar et al. (2021) also reported similar results for soy protein enrichment in instant noodles. Kinsella (1979) also advocated that the protein's shape is responsible for reduced viscosity. The intact, swollen starch granules are subjected to shear stress during the RVA holding period (95°C), which causes a loss of granule integrity. The resulting disruption of this biopolymer causes a decrease in paste viscosity. Thus, breakdown viscosity measures the material's resistance to shear and heat (Lim & Narsimhan, 2006). The WP significantly reduced starch granules disruption and showed the blend's substantially higher breakdown viscosity than the semolina alone (Khatkar et al., 2018a, 2018b), while both the samples exhibited lesser setback viscosity which is caused by rearrangement and reordering of the amylose chain when the sample cools to 50°C during the cooling phase. The final viscosity indicates the material's capacity to form a viscous gel or paste after cooking and cooling. It is the most important parameter in describing product quality. The final viscosity, directly related to the texture, was significantly reduced by adding SWP. Sudha et al. (2011) also reported decreased pasting properties by incorporating WP isolates.

### 3.3 Dough machinability

The RWP in semolina offered significant challenges during mixing and dough handling. RWP reduced the capacity of flour to absorb water, leading to inadequate mixing; thus, less water (28%) was required for mixing than the earlier reported (Khatkar et al., 2018a, 2018b) values for semolina (33%). RWP also produced a tough dough that was difficult to sheet and needed more time, energy, and effort than other counterparts. In general, it decreased machinability and resulted in rigid sheets of dough. Porwal et al. (2014) also stated an increase in dough development time with increased incorporation of chickpea flour into semolina. Sonication completely altered RWP's mixing and dough handling attributes. The addition of SWP increased WAC (31%) compared to RWP; thus, it improved mixing and handling. SWP increases the dough's softness and elasticity with ease in ball formation, resulting in smooth and specks‐free sheets. The dough's behavior was markedly noticeable throughout the process. The mixing and sheeting time was significantly reduced (about 4–5 times), thus improving the dough machinability (mixing–sheeting–handling). Consequently, more water was required to hydrate and flowable the material before mixing. Khatkar et al. (2021) and Du et al. (2016) also reported similar observation during dough handling for restructured soy protein and extruded SP isolates, respectively. This could be because proteins are amphiphilic, and modification exposes hydrophobic groups, encouraging disulfide and crosslink bonding for gluten. Overall, it improves dough's rheology and mixing behavior and provides a solution for mixing, handling, and sheeting protein‐enriched materials. In addition, it could also save costs for the food industry by reducing time and energy requirements.

### 3.4 Rheological properties

**FIGURE 1:**
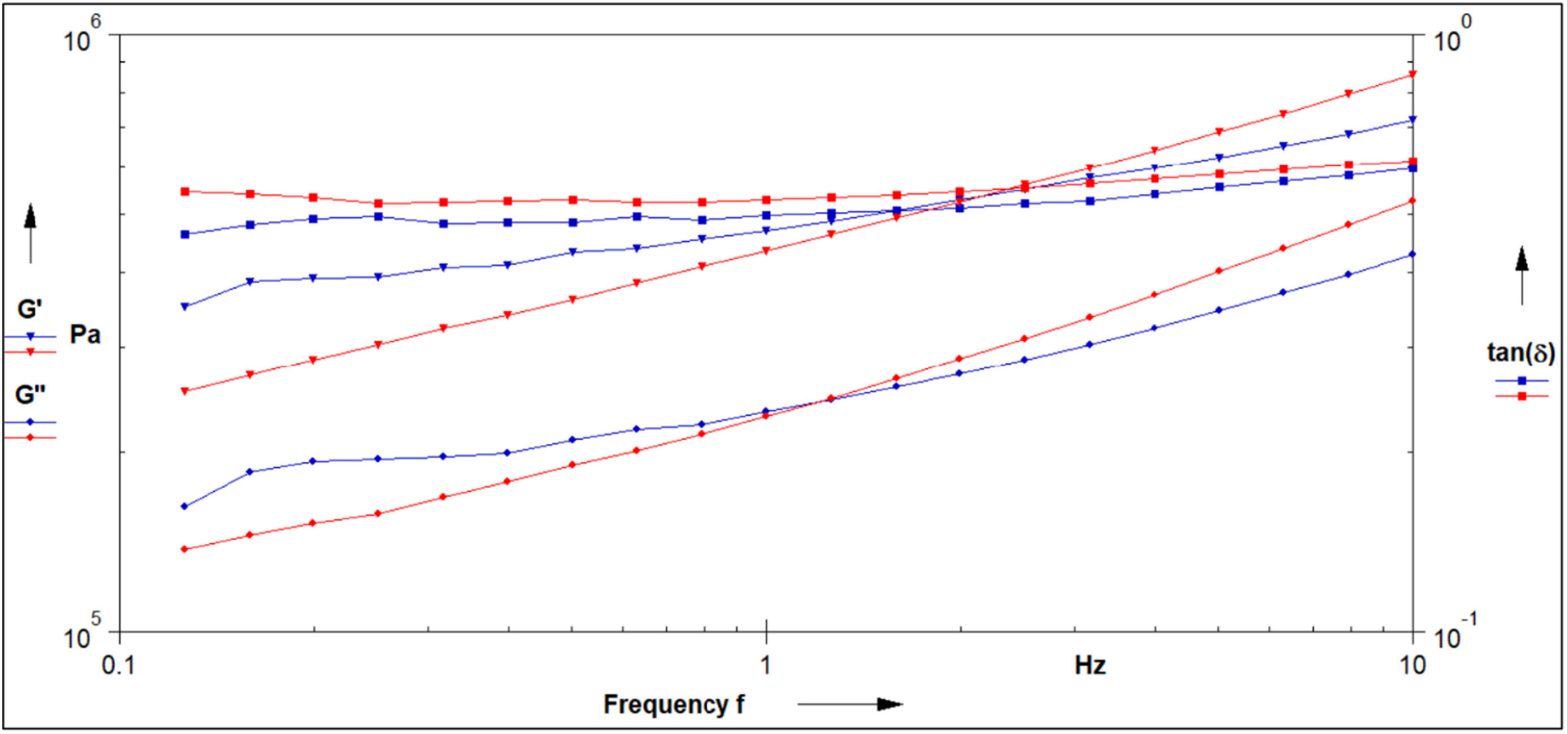
Rheological properties (storage modulus, *G*′; loss modulus, *G*″; and damping factor, tan *δ*) of SWP‐ (blue colored line) enriched instant noodles vis‐à‐vis RWP (red colored line)‐enriched instant noodles. RWP, raw whey protein; SWP, sonicated whey protein.

The storage (*G*′) and loss (*G*″) moduli represent elastic and viscous behavior, respectively. Both the samples exhibited an increasing trend against frequency (Hz) for *G*′ and *G*″ moduli (Figure 1) and acted like liquids (viscous) and solids (elastic) as of any viscoelastic structure. Since none of the samples showed higher adhesive properties than elastic behavior, the tan‐δ values are less than 1. Protein incorporation makes food rigid (Bhise et al., 2015; Khatkar et al., 2018a, 2018b, 2021).; therefore, RWP dough exhibited the same behavior, yielding higher solid properties than the control as reported earlier by Khatkar et al. (2021). This demonstrates the increased elastic modulus by adding RWP. RWP showed less viscous and elastic behavior on initial frequency than its counterpart, representing resistance for handling and sheeting. On the contrary, the SWP addition displayed higher storage and loss modulus values than the values for raw semolina (Khatkar et al., 2021). In contrast, at a higher frequency, SWP lowered the values without affecting tan‐δ, thus maintaining the appropriate dough rheology. It improved dough handling and smoothness by reducing loss and storage modulus. The dough with RWP and SWP showed good tan‐*δ* values, which justified its compactness and strength. Similar observations were observed during the dough preparation and mixing process, as discussed in Section 3.3. Dynamic oscillatory properties provide in‐depth knowledge of rheology and dough structure, and increased storage and loss modulus relates to improving gluten strength (Huang et al., 2010). Adding RWP and SWP increased the storage and loss moduli values proportionally and showed an increasing parallel trend. On the contrary, the raw semolina dough sample showed a significantly less pronounced increase with increasing frequency (Khatkar et al., 2021).

### 3.5 Cooking qualities

The cooking qualities results revealed that SWP had a significant (*p* < .05) effect on instant noodles' cooking characteristics (Table 2) and SWP significantly (*p* < .05) decreased cooking loss (7.48%) while considerably (*p* < .05) increased cooking weight (13.80%), water uptake ratio (14.35%), noodle diameter and expansion (4.47%), which were attributed to the intact structure by starch and SWP and their interactions. These properties are prominent for overall acceptability, economy, and industry. Simultaneously, it significantly (*p* < .05) increased cooking time within desirable limits and had industrial viability. The disruption of intramolecular electrostatic and hydrophobic interactions during ultrasonic cavitation caused the WP's helical structure to unfold, exposing hidden hydrophobic and hydrophilic groups (Khatkar et al., 2018a, 2018b; Zisu et al., 2010) and helping protein to interact within the starch matrix. Thus, SWP created a strong network in instant noodles, resulting in higher cooking time, water uptake ratio, and weight. The increased time for SWP noodles' cooking is due to higher WBC of SWP, which decreased water availability for starch particles, thus postponing and delaying swelling, gelation, and gelatinization process. However, the dense and firm network formation of starch (gelatinized) and protein (gelled) resulted in a decreased swelling volume of RWP noodles. The increased noodles diameters (mm) and per cent expansion of SWP noodles can directly be correlated with the water uptake ratio. The cooking results were better than restructured soy protein‐based instant noodles, as Khatkar et al. (2021) reported. Yeoh et al. (2014) also reported similar results for noodles enriched with cross‐linked SP isolates (microbial transglutaminase), which revealed a lower thickness and cooking loss, but fetched a higher hardness.

**TABLE 2:** Cooking and textural characteristics of SWP‐enriched instant noodles vis‐à‐vis RWP‐enriched instant noodles.

	Cooking qualities					Dimensional properties	
	Cooking time (min)	Cooking loss (%)	Cooking weight (%)	Water uptake ratio	Swelling index (%)	Noodles diameter (mm)	Noodles expansion (%)
RWP instant noodles	5.60 ± 0.36^a^	9.32 ± 0.22^a^	337.06 ± 16.70^a^	2.29 ± 0.04^a^	218.92 ± 19.03^a^	2.78 ± 0.05^a^	277.53 ± 5.43^a^
SWP instant noodles	6.18 ± 0.16^b^	9.62 ± 0.30^a^	336.32 ± 5.55^a^	2.39 ± 0.15^b^	213.58 ± 11.91^a^	2.85 ± 0.19^a^	284.87 ± 18.90^a^

	Textural characteristics						
	Hardness (*N*)	Adhesiveness (*N*.s)	Springiness (%)	Cohesiveness	Gumminess (*N*)	Chewiness (*N*)	Resilience
RWP instant noodles	47.02 ± 5.79^a^	−0.434 ± 0.042^a^	0.688 ± 0.039^a^	0.644 ± 0.059^a^	30.44 ± 6.24^a^	20.86 ± 3.70^a^	0.361 ± 0.070^a^
SWP instant noodles	57.56 ± 5.87^b^	−0.667 ± 0.033^a^	0.886 ± 0.092^b^	0.596 ± 0.036^a^	34.44 ± 5.59^b^	30.79 ± 7.64^b^	0.291 ± 0.033^a^

*Note*: Means (*n* = 3) with the same superscript in a column are not significantly different *p* < 0.05 from each other. The abbreviations RWP and SWP are for raw whey protein and sonicated whey protein, respectively.

### 3.6 Textural characteristics

SWP incorporation in noodles significantly (*p* < .05) increased the hardness, springiness, gumminess, and chewiness (Table 2). Instant noodles with SWP showed a decreasing trend, but not significantly, in resilience compared to RWP noodles and raw semolina noodles (Khatkar et al., 2018a, 2018b). The modified starch–protein interaction, which preserves structural integrity and increases hardness, is responsible for a better textural profile (hardness, springiness, cohesiveness, gumminess, and chewiness) of SWP noodles and enhances resistance to wear and tear throughout the supply chain. Similar results were also observed during dough handling and cooking instant noodles. The addition of soy protein to noodles (Khatkar et al., 2021; Yeoh et al., 2014); rice bran, egg albumin, and WPC (Phongthai et al., 2017); shrimp meat powder (Vijaykrishnaraj et al., 2015); egg (Ramya et al., 2015); and green mussel and fish powder (Desai et al., 2018) also increased pasta hardness.

### 3.7 Thermal features

The noodles containing RWP and SWP showed higher onset, peak, endset temperatures, and enthalpy (Table 3) compared to the values of semolina noodles, as reported earlier (Khatkar et al., 2018a, 2018b). The SWP addition significantly increased the peak temperature and enthalpy. The higher peak temperature revealed that the SWP noodles had a more ordered structure than the RWP noodles. The high *T*_p_ of SWP noodles resulted from a higher intermolecular interaction of modified protein and gelatinized starch, resulting in a stable structure, and requiring more energy for thermal transition. The protein and starch interaction also support higher enthalpy, and noodles need higher enthalpy for thermal transition, representing greater stability. The higher *T*_p_ and enthalpy values justify the longer cooking time of SWP noodles.

**TABLE 3:** Thermal features, *IVPD and* color characteristics of SWP‐enriched instant noodles vis‐à‐vis RWP‐enriched instant noodles.

	Thermal features				*IVPD*	Color characteristics		
	*T*_o_ (°C)	*T*_p_ (°C)	*T*_e_ (°C)	Δ*H* J/g		L*	a*	b*
RWP instant noodles	64.6	93.8	122.08	439.31	94.52 ± 0.45^a^	78.15 ± 0.27^a^	−2.05 ± 0.06^a^	12.26 ± 0.29^a^
SWP instant noodles	63.85	94.94	118.7	458.61	90.46 ± 0.34^b^	78.25 ± 0.29^a^	−0.24 ± 0.06^b^	13.45 ± 0.39^b^

*Note*: Means (*n* = 3) with the same superscript in a column are not significantly different *p* < .05 from each other. The abbreviations RWP, SWP, *T*_o_, *T*_p_, *T*_e_, Δ*H*, and *IVPD* are for raw whey protein, sonicated whey protein, onset temperature, peak temperature, end set temperature, enthalpy, and in vitro protein digestibility, respectively.

### 3.8 Determination of in vitro protein digestibility

Instant noodles' *IVPD* varied from 90.46% (SWP‐enriched noodles) to 94.52% (RWP‐enriched noodles) (Table 3) and much higher than the values of semolina alone (73.86%) noodles (Khatkar et al., 2018a, 2018b). The higher *IVPD* attributed to several factors such as greater digestibility, denaturation, structural unfolding, crosslinking, racemization and further degradation, aggregation, and re‐aggregation (Khatkar et al., 2021) of WP. However, the SWP‐enriched noodles delivered lower *IVPD* than RWP noodles due to the acoustic cavitation‐induced WP's structural unfolding that enhances intermolecular interaction during noodle preparation, thus resulting in fewer available sites for enzymatic hydrolysis. Similar results for *IVPD* were found for restructured soy protein instant noodles (Khatkar et al., 2021).

### 3.9 Microstructural properties

**FIGURE 2:**
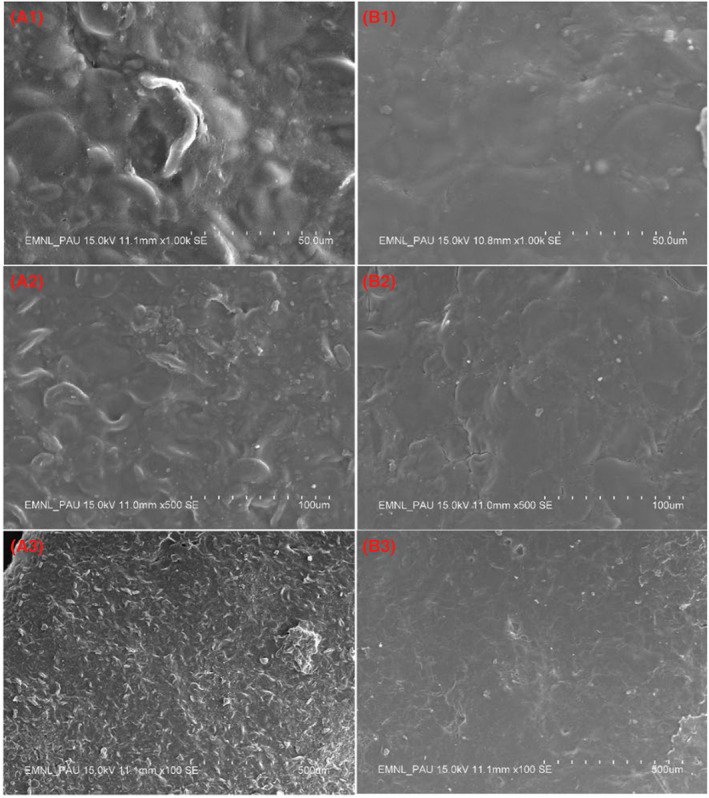
Scanning electron microscopy of RWP‐enriched (A1 at ×50, A2 at ×100, and A3 at ×500) and SWP‐enriched (B1 at ×50, B2 at ×100, and B3 at ×500) instant noodles. RWP, raw whey protein; SWP, sonicated whey protein.

The instant noodles' SEM images exhibited significant differences (Figure 2b,c). The gelatinized starch granules were embedded in the gluten network of the noodle, and no ditches and cracks were found on the surface (Figure 2) such conditions led to a tight/strong structure and minimizes the cooking losses. The texture of RWP noodles fetched a compact and filled surface with small gritty particles (Figure 2A1–A3). It revealed that RWP acted as a filler for semolina noodles but could not impart proper mixing and interaction with macromolecules; thus, it exhibited interaction only through surface adhesion. Tang and Liu (2017) reported similar results during the microstructure observation of wheat flour dough enriched with 10% and 30% WP. However, SWP noodles exhibited a smooth, compact, glossy surface without ditches and grittiness (Figure 2B1–B3) due to the smaller particle size, resulting in a larger surface area for interaction with gelatinized starch and wheat protein. The intermolecular interaction and the compact surface can also be confirmed with higher cooking time and textural attributes of SWP noodles. The resultant strong network of SWP with gluten protein covers the gelatinized starch completely. SEM image verified the significant influence of SWP on microstructure and confirmed the adequacy of SWP on functional, cooking, textural, and sensory characteristics. The results are like those of Yeoh et al. (2014) and Khatkar et al. (2021), who reported improved microstructure of modified soy protein noodles.

### 3.10 Color and sensory characteristics

There was no significant (*p* > .05) difference in the L* value of RWP and SWP noodles (Table 3) but, SWP noodles exhibited significantly (*p* < .05) lesser a* (0.24 ± 0.06) and b* (13.45 ± 0.39) values than RWP noodles. Therefore, in contrast, SWP noodles also displayed a desirable yellow tinge, contributing to the improved surface appeal. Similar results for a* and b* variations after incorporation of the raw and modified protein (soy) were reported by Du et al. (2016) for Chinese steamed bread and by Khatkar et al. (2021) for instant noodles. The results of the sensory attributes are presented in Table 4. Regarding surface appeal, hardness, stickiness, biting behavior, mouthfeel, and overall acceptability, the SWP noodles scored significantly (*p* < .05) higher scores. The judges preferred the lightness (slightly yellowish), surface appeal, textural integrity, and smoothness of SWP noodles over other samples. However, SWP powder is whiter than RWP, but the prepared noodles were still slightly yellow which might be due to the Maillard browning between modified protein and carbohydrate. The SWP noodles were tastier and highly acceptable than RWP and control noodles. The significant increase in the quality attributes and sensory of SWP noodles supports the suitability of protein modification for value addition in instant noodles. Similar results for sensory attributes were also reported for restructured soy protein‐enriched instant noodles (Khatkar et al., 2021).

**TABLE 4:** Sensory profile of SWP‐enriched instant noodles vis‐à‐vis RWP‐enriched instant noodles.

	Surface appeal	Hardness	Stickiness	Biting behavior	Mouthfeel	Overall acceptability
Control instant noodles	7.40 ± 0.52^a^	7.55 ± 0.37^a^	7.60 ± 0.32^a^	8.05 ± 0.50^b^	7.50 ± 0.53^a^	7.62 ± 0.16^a^
RWP instant noodles	7.85 ± 0.82^ab^	7.40 ± 0.61^a^	7.85 ± 0.34^ab^	7.25 ± 0.92^a^	7.40 ± 0.94^a^	7.55 ± 0.37^a^
SWP instant noodles	8.15 ± 0.50^b^	8.55 ± 0.71^b^	8.25 ± 0.57^b^	8.25 ± 0.83^b^	8.20 ± 0.81^b^	8.28 ± 0.44^b^

*Note*: Means (*n* = 3) with the same superscript in a column are not significantly different *p* < .05 from each other. The abbreviations U‐WP IN and S‐WP IN are for raw whey protein and sonicated whey protein, respectively.

## 4 CONCLUSION

Modifying WP by sonication is a promising technique to add value to instant noodles. Sonication significantly overcomes the technological challenges associated with protein fortification. The SWP improved the dough machinability, and even with less time (about 4–5 folds), the sheets were smooth, more favorable, and free of specks. Dough exhibited a viscoelastic structure with higher values of elasticity as observed by storage and loss moduli. The SWP significantly improved the prominent properties of industry and consumer concerns. It decreased cooking loss (7.48%) and increased cooking weight (13.80%), water uptake ratio (14.35%), noodle diameter (4.47%), along with hardness, springiness, gumminess, chewiness, and enthalpy. SWP provided a compact and ordered structural morphology to instant noodles with resistance to tear and wear while handling, improved *IVPD*, surface appeal, and overall acceptability. Overall, the study offers SWP as a quality protein ingredient for stability and mass fortification in high‐heat processed foods, which can easily overcome technical hitches linked with protein enrichment.

## AUTHOR CONTRIBUTIONS

**Anju Boora Khatkar:** Conceptualization (equal); data curation (equal); investigation (equal); methodology (equal); validation (equal); writing – original draft (equal). **Amarjeet Kaur:** Conceptualization (equal); supervision (equal); validation (equal); writing – review and editing (equal). **Sanju Bala Dhull:** Supervision (equal); writing – review and editing (equal). **Sunil Kumar Khatkar:** Data curation (equal); methodology (equal); software (equal); writing – review and editing (equal). **Nitin Mehta:** Resources (equal); writing – review and editing (equal). **Jaspreet Kaur:** Methodology (equal); validation (equal). **Gulden Goksen:** Supervision (equal); writing – review and editing (equal).

## CONFLICT OF INTEREST STATEMENT

The authors declare that they have no known competing financial interests that could have appeared to influence the work reported in this paper.

## Data Availability

The data that support the findings of this study are available from the corresponding author upon reasonable request.
